# High-grade serous ovarian cancer: the clone wars

**DOI:** 10.1007/s00404-017-4292-1

**Published:** 2017-02-03

**Authors:** Aleksander Salomon-Perzyński, Magdalena Salomon-Perzyńska, Bogdan Michalski, Violetta Skrzypulec-Plinta

**Affiliations:** 1 Department of Internal Medicine and Oncological Chemotherapy, School of Medicine in Katowice, Medical University of Silesia, Katowice, Poland; 2 Department of Gynaecology Oncological, School of Health Sciences in Katowice, Medical University of Silesia, Katowice, Poland; 3 School of Health Sciences in Katowice, Medical University of Silesia, Katowice, Poland

**Keywords:** Ovarian cancer, Clonal evolution, Drug resistance, Genetic heterogeneity, Genomic instability

## Abstract

**Background:**

The last 5 years’ studies using next-generation sequencing provided evidences that many types of solid tumors present spatial and temporal genetic heterogeneity and are composed of multiple populations of genetically distinct subclones that evolve over time following a pattern of branched evolution. The evolutionary nature of cancer has been proposed as the major contributor to drug resistance and treatment failure. In this review, we present the current state of knowledge about the clonal evolution of high-grade serous ovarian cancer and discuss the challenge that clonal evolution poses for efforts to achieve an optimal cancer control.

**Methods:**

A systemic search of peer-reviewed articles published between August 2007 and October 2016 was performed using PUBMED and Google Scholar database.

**Results and conclusions:**

Recent studies using next-generation sequencing have allowed us to look inside the evolutionary nature of high-grade serous ovarian cancer, which in the light of current evidence can explain the relapsing course of the disease frequently observed in the clinical practice. Since only minimal improvement in the survival of patients treated with standard therapy has been observed in the last decade, novel molecular targeted therapies are of great interest in high-grade serous ovarian cancer. However, both spatial and temporal intratumoral genetic heterogeneity is a major challenge for personalized medicine, and greater knowledge of the molecular rules that drive tumor evolution through space and time is required to achieve a long-term clinical benefit from personalized therapy.

## Introduction

High-grade serous ovarian cancer (HGSOC), the most common and aggressive form of the epithelial ovarian cancer (EOC), remains the leading cause of cancer-related death among all gynecological cancers in the developed parts of the world [1, 2].

The majority of cases show a significant, but transient response to standard therapy including debulking surgery followed by platinum-based chemotherapy, and the development of resistance is almost permanently inscribed in the clinical course of the disease [3]. Therefore, the emergence of drug-resistant disease is a major problem in the clinical management of HGSOC, and in the context of the still unsatisfactory treatment outcomes, deciphering the molecular mechanisms that contribute to drug resistance is the greatest challenge in the area of HGSOC molecular research.

The last 5 years’ studies using next-generation sequencing provided evidences that many types of solid tumors present spatial and temporal genetic heterogeneity and are composed of multiple populations of genetically distinct subclones that evolve over time following a pattern of branched evolution in a similar manner to the Darwinian evolution of species [4–9]. This evolutionary nature of cancer has been proposed as the major contributor to drug resistance and treatment failure [10].

In this review, we present the current state of knowledge about the clonal evolution (CE) of HGSOC and discuss the challenge that CE poses for efforts to achieve an optimal cancer control.

## Genomic instability in HGSOC

Large-scale genomic analyses demonstrated that HGSOC exhibits a high degree of genomic instability (GI) arising as a result of DNA repair defects caused mainly by TP53 mutations and homologous recombination (HR) deficiency, occurring in 96% and almost 50% of cases, respectively [11, 12]. During tumorigenesis GI promotes the acquisition of further DNA alterations leading to genetic diversity between cancer cells and creating the possibility of coexistence of genetically distinct subclones within the same tumor [13]. Therefore, GI is a source of intratumoral genetic heterogeneity (ITH) and one of the most important driving forces of CE. Subclones can benefit from GI by acquiring new genomic events that confer a selective advantage during ongoing evolution, thus a high level of GI is generally associated with emerging of treatment resistance and poor prognosis in various cancer types [14–16]. However, a high degree of GI can also have an unfavorable effect on the fitness of cancer subclones by enabling the acquisition of deleterious genomic alterations that provide a selective disadvantage [17] and, consequently, limit tumor growth, and/or increase tumor response to the cytotoxic therapy. This is particularly true for HGSOC, where the studies have shown that a greater level of GI is associated with improved outcomes, mainly due to a higher response rate to platinum-based chemotherapy [18, 19]. It is consistent with clinical observations that HR-deficient cases with a highly unstable genome exhibit enhanced platinum sensitivity and improved overall survival (OS) compared to HR-intact cases [20]. Since HR deficiency is associated with better response to platinum agents, the restoration of BRCA1 and BRCA2 function is thought to play an important role in emerging of platinum resistance [21]. Recently, the large-scale genomic analysis of chemoresistant HGSOC, showed HR deficiency only in 2 out of 12 platinum-refractory cases (both as a consequence of the somatic methylation of BRCA1 which cannot be considered as an equivalent to germline mutation) while reversions of germline BRCA1/2 mutations have been found in 5 out of 10 relapse cases. Moreover, one autopsy case was found to have several independent subclonal BRCA2 reversion events detected in the different tumor metastatic sites at the time of relapse [12]. Therefore, it appears that in some HGSOC cases an optimal level of GI may be required to provide cancer subclones with the ability to survive and to expand under selective pressure of chemotherapy. Further instability, may in turn be unfavorable for subclones leading to their increased chemosensitivity. This hypothesis is supported by a recent report demonstrating an increase in genomic stability within the residual subclones after the course of neoadjuvant chemotherapy [22]. Further studies should evaluate how various levels of GI, which are seen in HGSOC, affect the ITH, the tumor evolutionary potential and, ultimately, the tumor response to the treatment.

## Clonal evolution of pre-treatment disease

To investigate clonal heterogeneity in HGSOC, Bashashati et al. obtained 29 spatially separated samples from 5 patients with newly diagnosed stage III–IV HGSOC [7]. Exome sequencing, copy number analysis, target amplicon deep sequencing and gene expression profiling confirmed the presence of extensive intratumoral genomic and transcriptomic heterogeneity with TP53 mutation as the clonally dominant key driver event acquired early in the tumorigenesis. Importantly, alterations in key driver genes such as PIK3CA, CTNNB1, PDGFR, NF1, SH3GL1, RBM15 were found to be subclonal, indicating that they have been acquired during tumor evolution and confirming that key driver events contributing to cancer initiation, progression and maintaince are not always clonally dominant (present in all cancer cells), but also can be present subclonally (present only in a subset of cancer cells) [4, 5, 23]. These subclonal mutations along with non-genetic factors can lead to a phenotypic diversity between cancer cells and provide the fitness advantage to subclones, reducing clinical benefit of cancer therapy [23, 24]. Indeed, some of the subclonal mutations found by Bashashati et al. were associated with alteration in gene expression profile supporting their role in shaping subclonal phenotypes and intratumoral phenotypic heterogeneity.

The results reported by Hoogstrat et al. [25] highlighted that the patterns of CE vary across HGSOC cases. These findings also suggest that CE may occur independently at the level of mutational processes as well as genomic rearrangements, and alterations in genomic rearragements may, independently from mutations, affect the intratumoral phenotypic diversity. They took 15 spatially separated samples from different tumor sites from 2 treatment-naive patients with stage IIIC–IV HGSOC. The first case showed extensive genomic and transcriptomic intratumoral heterogeneity with most marked differences in the genomic rearrangement, the gene expression profile and key cancer pathways activation between the samples from the primary tumor site in the ovary and those obtained from peritoneal and omental metastases. However, the second case was found to be much more homogenous with respect to genomic rearrangement, mutational profile and gene expression pattern. Importantly, in the first case there were no mutations unique to metastatic samples and all mutations identified in these samples were also found in samples from the right ovary. In the second case only two mutations were unique to the tumor metastatic site, whereas all other detected mutations were shared between all samples. This supports the results from the Lee et al. [26] case report and suggests that in some cases of pre-treatment HGSOC, intraperitoneal metastases may arise with only a little accumulation of new somatic mutations.

Studies showed a rather consistent picture of HGSOC as a dynamic entity composed of multiple populations of genetically and phenotypically distinct subclones evolving from a single ancestral clone following patient-specific patterns of branched evolution [7, 25–27]. In the context of complex tumor structure it is most likely that the CE of untreated HGSOC is mainly driven by selective pressures imposed by highly heterogeneous (both spatially and temporally) tumor microenvironment (TME) [28, 29]. During ongoing evolution, subclones are selected according to their fitness to survive in divergent microenvironmental conditions [29, 30]. Selection is based on the phenotypes and subclones that have phenotypic advantage in given environmental landscape undergo further clonal expansion [31]. Phenotypes are not, however, the permanent features of cancer cells and do not result solely from cell-autonomously acting factors [10, 32, 33]. Beyond genetic, epigenetic, transcriptomic and proteomic factors, also variations in local microenvironmental niches may affect phenotypes of cancer cells and consequently influence their fitness [10, 24, 29, 34]. These interactions are not, however, directed unilaterally and cancer cells can likewise modulate their microenvironments enforcing, e.g., dynamic changes in their own phenotypes [24, 35]. Therefore, the TME should be considered not simply as a “passive" source of various selection forces that promote certain phenotypes, but rather as a dynamic, complex structure actively affecting the pathways of cancer cell evolution.

It appears that in an advanced-stage HGSOC the presence of extensive phenotypic diversity as well as noticable differences in chemosensivity between cancer cells isolated from different tumor deposits [36] can be in part explained by divergent selective pressures acting on tumor cell subpopulations in various metastatic niches. As clinical observations indicate that in advanced-stage EOC the initial disease distribution is prognostically relevant regardless of achieving a cytoreduction to microscopic residual disease (RD) [37], the role of microenvironmental conditions in given metastatic regions in selection or “storage” of resistant subclones leading to tumor maintaince and progression should be evaluated in further studies. There is also a need to assess whether the metastatic niches in the given metastatic organ are able to promote repeatable genotypes and phenotypes across different HGSOC cases.

The coexistence of genetically dissimilar subclones widespread within the three-dimensional (3D) tumor space can lead to interclonal interactions which are not limited, however, to a simple competition for space and resources during ongoing selection [32, 38–40]. As a recent study using mouse xenograft model of breast cancer suggests, the heterogeneous cancer cell population may include minor subclones too indolent to win a competition and grow out/expand, but able to promote proliferation of other subclones [32]. Along with the results of the study in glioblastoma [38], it supports the potentially relevant role of minor subclonal populations in driving cancer growth and maintaining tumor heterogeneity. Moreover, a study based on transgenic mouse model of multiple myeloma suggests that the inability of minor subclones to compete efficiently does not necessarily lead to their exclusion from the cancer cell population by more agressive subclones. Dominant subclones may indeed supress minor subclones, but they also may coexist with them or even promote their proliferation [40]. These findings shed new light on the role of interclonal interactions in cancer evolution and provide additional evidence to perceive tumor as a complex topological “ecosystem” rather than a simple mass of transformed epithelial cells. Recently, several studies revealed that 3D models of EOC cell lines that reconstitute complex tumor architecture better reflect cancer cells behavior and their potential for emergence of resistance than two-dimensional models [41–43] supporting the impact both cell–cell and cell–stroma interactions on EOC biology. Due to the subclonal complexity of HGSOC, further studies should evaluate whether interclonal interactions have a relevant effect on tumor evolution prior to the treatment, on tumor response to the treatment, as well as on the development of platinum-resistant and, especially, platinum-sensitive recurrence [44].

## HGSOC evolution during the course of treatment

The emergence of treatment resistance followed by initial response to standard therapy resulting in tumor relapse remains a main clinical problem in the management of most HGSOC cases reducing the possibility to cure the advanced-stage disease [3, 45].

For heterogeneous tumor cell population, cancer therapy constitutes a selection pressure widely affecting the patterns of tumor evolution [10]. Beyond mechanical and/or cytotoxic eradication of sensitive subclones, treatment-related selection forces can also favor the expansion of subclones genetically and/or phenotypically best adapted to therapy-induced conditions leading to dynamic changes in the subclonal composition of cancer [46–48]. It should be noted, however, that because of the existence of a structural complexity of the tumor, the adaptive changes in the tumor subclonal architecture that occur during the course of treatment probably are not determined solely by the direct effect of cancer therapy on cancer cell vitality, but also by its impact on TME [49], and by its interference into the clonal competition or, in a broader sense, into the interclonal interactions [48].

HGSOC evolution over the course of treatment have been analyzed in several studies. Recently, paired tumor samples taken before and after first line of platinum-based chemotherapy have been compared using whole-exome sequencing (WES) and single nucleotide polymorphism profiling [50]. While only 58% of somatic mutations were conserved between matched tumor biopsies, 27 and 15% of them were found to be relapse- and primary-unique, indicating the existence of substantial genetic heterogeneity between primary and recurrent tumors. The majority of tumor pairs demonstrated complex clonal dynamics, with some of the subclonal mutations increasing and another decreasing in frequency between primary and relapse samples. Although all but four biopsies contained subclonal mutations, its frequency was relatively low suggesting a rather oligoclonal than policlonal nature of HGSOC. It should be emphasized here that the clonality analysis of a single tumor biopsy is restricted to the biopsy taken for analysis; therefore, it does not reflect the full spectrum of tumor subclonality (sampling-bias) [51]. Moreover, currently used sequencing strategies have a limited ability [52] to detect low prevalent subclones, hence a real subclonal complexity of HGSOC may be underestimated. Therefore, further studies should use more precise sequencing strategies, such as a single cell sequencing, able to identify low prevalent subclones and, consequently, providing a full insight into the spatial subclonal composition of HGSOC and its evolution over time [53].

Branched CE in the progression of HGSOC from primary to reccurent disease has also been indicated by a case study using WES and comparative genome hybridisation to compare samples taken during debulking surgery first at initial diagnosis and second at disease relapse after treatment with platinum-based chemotherapy in combination with bevacizumab [54]. Only 42 out of 102 somatic mutations were common to all samples collected whereas 21/102, 10/102 and 7/102 were unique to biopsies obtained from primary tumor, intra-pelvic and extra-pelvic recurrence, respectively. Even lower levels of concordance between the primary and the relapsed disease have been reported recently by two other studies using targeted re-sequencing technology to compare mutational landscape in terms of 65 selected pharmacologically relevant genes between tumor samples taken before and after treatment with at least one line of chemotherapy [55, 56]. As one of these studies showed, the clonal architecture of recurrent tumors was more homogeneous than their primary counterparts suggesting that during ongoing evolution the majority of the somatic mutations were eliminated from cancer cell population by selective forces imposed by cancer therapy [56].

Studies provided consistent evidence that HGSOC continues evolution during the course of treatment following highly individual patterns of CE [50, 54–56]. To date, however, little is known about how the differences in the evolutionary potentials of the tumors affect the clinical outcomes. In a recent paper, 135 samples from 14 patients with advanced-stage HGSOC, who received platinum-based chemotherapy, were analyzed to evaluate the relationship between ITH and survival [27]. ITH was quantified as the degree of clonal expansion using the novel MEDICC (Minimum Event Distance for Intra-tumour Copy Number Comparisons) algorithm. As expected, the degree of clonal expansion differed considerably between patients, confirming earlier conclusions that HGSOC exhibits patient-specific ITH [7, 25, 50]. Importantly, patients with higher clonal expansion had shorter progression-free and OS compared to those with low clonal expansion, suggesting that highly heterogeneous polygenomic tumors have a greater predisposition to acquire treatment resistance, and, therefore, are characterized by poorer outcomes.

All studies that have monitored HGSOC evolution over the course of treatment consistently suggest that tumor relapse originates from drug-resistant subclone/s originally present in the primary tumor that expand under selective pressure of therapeutic intervention [27, 50, 54–56]. In contrast to some other cytotoxic drugs, such as temozolomide [57], there are no evidences that the mutagenic activity of platinum could result in the generation of resistance de novo [12, 27, 50], implying that the role of platinum-based chemotherapy in the arising of treatment resistance in HGSOC is limited to the selection of already present resistant subclones.

The fact that in all cases adjuvant chemotherapy failed to destroy resistant subclones [27, 50, 54–56] highlights that the primary cytoreductive surgery carried out precisely is essential in the management of HGSOC. From the HGSOC heterogeneity point of view, malignant lesions should be removed to the greatest extent possible to achieve long-term clinical benefits from the applied therapy. Otherwise, minor resistant subclonal populations preoperatively widely distributed in tumor space may persist in the RD contributing to the rapid development of chemoresistance. It is clearly reflected by clinical observations indicating that the amount of RD left after primary surgery is a major prognostic factor for survival in patients with EOC and the probability of emergence of resistance increases and the time to resistance decreases with the volume of RD [58, 59]. Since RD should be regarded as a reservoir of resistant subclones, the current efforts to reduce its amount by allowing resection of additional malignant tissue that normally remains invisible during surgery represent a very promising way to optimize therapy [60].

## Conclusions and future directions

Advances in sequencing techniques have allowed us to look into the evolutionary nature of HGSOC, which in the light of current evidences can explain the relapsing course of the disease observed in clinical practice. Since only minimal improvement in the survival of patients treated with standard therapy has been observed in the last decade, novel molecular targeted therapies are of great interest in HGSOC [61]. However, both spatial and temporal ITH pose a major challenge for personalized medicine [62], and greater knowledge of the molecular rules that drive tumor evolution through space and time is required to achieve a long-term clinical benefit from personalized therapy.

Since growing amount of evidence suggests that HGSOC relapse arises from outgrowth of pre-existing drug-resistant subclonal populations, further integrative genomic and phenotypic analyses using precise sequencing techniques should be carried out to define the molecular and genotypic signatures of resistant subclones. These data, respectively cataloged, could be used to evaluate the differences in the resistance patterns between individual patients and also could serve as a starting point for the design of novel therapeutic strategies. Further pre-clinical studies should give answer to a number of intriguing questions: (1) when do resistant subclones arise during the HGSOC evolution? (2) Whether they are distributed randomly or stochastically within the primary tumor? (3) What types of functional relationships do link them with dominant clones and surrounding non-malignant cells? (4) What is the role of microenvironmental niches in their selection or storage? (5) Whether their genotypes or phenotypes have a decisive influence on their positive selection? (6) Whether they are able to lead to recurrence autonomously or need support from the surrounding cells?

Personalized medicine requires tools to provide precise data on the subclonal composition of a patient’s tumor at the time of diagnosis, and which will also allow for regular tracking of its changes in relation to the therapeutic intervention [63]. With the knowledge of how the subclonal composition of tumor changes under therapy, we will be able to set the combined or sequential treatment that can prevent selection of resistant subclones [64]. Therefore, more emphasis should be placed on the improvement of non-invasive approaches like the circulating plasma cell-free DNA sequencing whose usefulness in determining HGSOC subclonality has been recently demonstrated [65, 66].

Although existing evidences suggest that HGSOC displays highly individual patterns of CE, they are based on a relatively small number of cases. Therefore, further genomic analyses on representative groups of cases at different clinical stages should assess to what extent patterns of evolution are reproducible between patients and to what extent they are predictable in individual patients.

Heterogeneity is a hallmark of HGSOC. It must, therefore, be taken into account during efforts to improve efficiency of standard therapy, as well as during design of novel personalized therapeutic strategies.
